# Baseline Assessment of Primary Healthcare Delivery Through Subcenters of Northern India

**DOI:** 10.7759/cureus.6391

**Published:** 2019-12-16

**Authors:** Sonu Goel, Madhur Verma, Kamal Kishore, Kritika Upadhyay, Vijai Sharma

**Affiliations:** 1 Epidemiology and Public Health, Post Graduate Institute of Medical Education and Research, Chandigarh, IND; 2 Community and Family Medicine, All India Institute of Medical Sciences, Bathinda, IND; 3 Biostatistics, Post Graduate Institute of Medical Education and Research, Chandigarh, IND

**Keywords:** national health mission, subcenters, supportive supervision, universal health coverage.

## Abstract

Background

Our study assessed the status of facilities and services available at the subcenter level and identified the gaps that deter the realization of universal health coverage.

Methods

A cross-sectional observational study design was used for assessing the subcenters of the Ambala district, Haryana, India with a predesigned, semi-structured tool containing 88 items marked on an ordinal scale. The subcenters were assessed and scored as per their functioning and delivery of various services, their coverage, and quality in terms of various national health programs.

Results

We found that the essential infrastructure of most of the buildings was average. The types of equipment for antenatal examination, vital medicines, and prominent display boards in the local language were present in all subcenters. The majority of the health workers (n = 27; 93%) successfully demonstrated proper handwashing techniques; 65% correctly measured blood pressure during an objective structured clinical examination (OSCE), and 48% could correctly estimate hemoglobin with a hemoglobinometer. Sound knowledge regarding maternal and child healthcare practices and guidelines was noted in 89% of the health workers.

Conclusions

The health system of Haryana has scope for improvement in terms of health centers. It is pertinent for the realization of universal health coverage. Therefore, internal audits similar to what we performed must be planned and executed as a regular activity within the framework of existing health systems.

## Introduction

India is currently experiencing a rapid transition from communicable to non-communicable diseases, with some evident overlapping [1]. To effectively combat these problems and achieve universal health coverage, service delivery must be improved at the grass-roots level. The Kartar Singh Committee put forward the concept of subcenter (SC) in 1973. The committee proposed the division of primary health centers (PHC) into 16 SCs, to be managed by a team of one male and one female health worker [2]. The Government of India accepted these recommendations and implemented them in the fifth 5-year plan. The National Health Policy, 2017 recommended strengthening the delivery of primary healthcare through the upgrading of the existing SCs and establishment of health and wellness centers as a platform to deliver comprehensive primary healthcare and called for an allocation of two-thirds of the health budget to primary healthcare [3].

The Indian Public Health Standards (IPHS), revised in 2012, lays down the essential and desirable requirements for services, infrastructure, equipment, human resources, and drugs for public health facilities at the SC level [4]. The resources in terms of infrastructure, human resources, and logistics are critical determinants of the service quality delivered by a particular SC. For ethical practice and patient safety, rules, standards, and notifications must be applied to all sectors uniformly. The SCs are constantly criticized for their inability to provide quality health services due to various reasons [5]. In Haryana alone, a shortfall in SCs by approximately 23% was recently estimated [6].

Supervision was recognized as the most crucial step in improving the SC status and achieving universal health coverage [7]. While supervision can be interactive, traditional supervision focuses on the inspection and discovery of faults instead of problem-solving to improve performances [8]. Health workers often receive little advice or guidance on how to improve their results. They are often left unsupervised without a clear definition of objectives. Sometimes the supervisors do not have the technical, managerial, and supervision skills required to assess health facilities adequately. Therefore, they are unable to provide professional advice and appropriate feedback to improve service delivery. Another type of supervision is supportive supervision (SS). It is defined as “a process that promotes quality at all levels of the health system by strengthening the relationships within that system, with an emphasis on identifying and solving problems and contributing to the optimization of the allocation of resources-promotion of high standards, teamwork and better communication in both directions” [9]. The SS creates an enabling environment, facilitates two-way communication, and builds team approaches that improve problem-solving [10]. SS ensures that the tasks are being implemented correctly, focusing on goals and using data optimally for decision-making.

Such supervision leads to an increased sense of ownership, increased work satisfaction, decreased attrition rates, and overall increased work performance among health workers [11]. However, due to the shortage of doctors in our country, SS was neglected until the past few years. Furthermore, it had limited and intermittent activity. SS had been an integral activity in the state of Haryana for some time, but the results were not documented. Moreover, SS was limited to certain domains of the health system, and health facilities were not assessed comprehensively. We have been regularly implementing SS activities in our study area for the past few years. In the present study, we intend to present results from the baseline assessment of the SCs and highlight the problems observed in the infrastructure and service delivery.

## Materials and methods

Study settings

Haryana is one of the northern states of India, with approximately 25 million inhabitants spread across 22 districts [12]. Each district has three to four Community Development Blocks, with each block catering to approximately 80,000-100,000 people. The state health system delivers its services with the help of 2,630 SCs, 486 PHCs, 119 community health centers (CHCs), and 21 district-level hospitals [13]. Antenatal care and immunization services are offered free-of-charge at all levels according to national guidelines. However, Haryana is the only state in India with the dubious distinction to have recorded a rise in maternal deaths in the past two decades [14]. The present assessment was done in Shahzadpur block of Ambala district, which is a field practice area of the Department of Community Medicine and School of Public Health, Post Graduate Institute of Medical Education and Research (PGIMER), Shahzadpur block, Chandigarh. The government health facilities of this area include a CHC, four PHCs, and 29 SCs.

Study design and sampling

The cross-sectional observational study design was adopted to conduct the study from January to December 2017. Universal sampling was used, wherein all the SCs (n = 29) in the study area were included in the study.

Study tool

A predesigned, semi-structured tool with 88 items and different questions grouped into 11 broad domains was used in this study. The tool collected information on the infrastructure, service delivery, service utilization, quality of services, and operational issues. The tool was adapted from the initial SS tools used by the national health mission department of Haryana. It was further modified by a faculty and two residents of Community Medicine, School of Public Health, PGIMER, Chandigarh. The tool was pretested in two SCs situated in a different area under the department. Minor changes were made, and the tool was finalized; content validation was done by experts from the district administration, such as a Chief Medical Officer of the Ambala district, Block Medical Officer of Shahzadpur, a lady health visitor, and faculty from PGIMER, Chandigarh. The service quality was determined objectively by assessing the clinical competency of the male and female health workers posted at SC with the help of the objective structured clinical examination (OSCE). The OSCE is a versatile multipurpose assessment method for appraising healthcare providers. It evaluates competency based on objective testing through direct observation. Originally, the examination comprises several “stations” in which examinees are expected to perform several clinical tasks within a specified period against specific predetermined criteria essential to demonstrate adequate competence for that particular clinical skill. Moreover, the OSCE evaluates areas that are pertinent to the optimal performance of healthcare providers, such as their ability to record/interpret data, problem-solving, teaching, communication, and handling unpredictable patient behavior, which is otherwise impossible in the traditional clinical examination [15].

Study Protocol

The final tool was shared with district authorities in advance before undertaking the study. Data were collected as a part of regular SS visits in the health centers of the study area by the residents posted in the Department of Community Medicine. The residents were MD students and had a basic understanding of the hierarchy of public healthcare systems in India and of the IPHS guidelines. The protocol of SS visits to health centers was discussed with them in detail, and they were then asked to gain more information about the OSCE. The primary investigator demonstrated the OSCE process during pilot testing so that they felt comfortable with it. After that, a formal schedule for SS visits was communicated with the district health administrators for necessary permissions, and the tour was planned such that it did not disturb the routine work schedule of the centers. Moreover, the schedule was shared with the auxiliary nurse-midwife (ANM) in charge of the SC and the medical officers of the concerned PHC. On the day of the visit, the residents first collected the baseline information about the number of beneficiaries and the logistics situation of the SC. This was followed by a knowledge assessment of the health workers on topics like danger signs of a newborn, high-risk pregnancy, basic treatment guidelines, basket-of-choice approach for family planning, national health programs, and potential beneficiaries. The OSCE assessment was done for different services such as blood pressure (BP) measurement, hemoglobin (Hb) estimation, and protein and albumin content of urine.

Statistical analysis

The baseline data were entered twice in MS Excel 2013 version (Microsoft, Redmond, WA) and analyzed in the statistical package for social sciences (SPSS) for Windows version 16.0, 2008 version (SPSS Inc., Chicago, IL). All the data collected were validated. It was cross-verified by the principal investigator periodically. Descriptive characteristics were comprehensively assessed and reported in terms of counts, frequencies, and means value as appropriate. Each item was marked on an ordinal scale (good, average, or poor) that measured its effectiveness. Operational definitions were set to label the tool item as good, average, and poor beforehand. If the service or records were well maintained and up-to-date, they were graded as good, if the documents were maintained but not up-to-date, they were marked as average, or otherwise marked poor. The essential skills of health workers were rated based on the scores obtained through OSCE assessment and were labeled as good, average, and poor if the total score obtained was 80%-100%, 50%-80%, and <50%, respectively. The SCs were evaluated and scored as per the delivery of various national health program services, coverage, and service quality. For this, questions under each domain were assigned one mark. They were then averaged to get a mean score for each domain. This helped us depict the overall performance of the SCs for that domain.

## Results

Demographic characteristics

A total of 29 SCs were assessed during the study period. The mean population catered by each SC was 7,112.5 ± 1,552.1 (range: 5,042-11,820), the mean number of pregnant women enrolled in the SC was 128.07 ± 39.45, and the average birth rate in the past financial year was 17 ± 3.49. Out of the total two job positions of ANMs at the SC, the first one was hired permanently, while the other one was on a contractual basis. It was observed that contractual job positions were filled in most of the SCs (n = 28), while only 50% (n = 1 5) SCs had their regular positions filled. Only four (13.8%) regular positions of multi-purpose health worker-male (MPHW-M) were filled against the sanctioned post of 29. All villages had accredited social health activists (ASHAs). The mean distance of SC from its PHC was 8.6 ± 5.36 km (range: 0-20 km).

Health facility infrastructure and basic facility assessment

The building condition was average in 15 (52%) of 29 SCs (Table 1). In total, 12 (41%) centers had two rooms, but only one room was being used to provide services. Round-the-clock water supply was present in most of the SCs, but it was not potable, and most of the centers depended on their neighbors. Toilets were non-usable in five (17%) centers. Most of the centers (n = 18; 62%) had no provision for uninterrupted electricity supply. Equipment for antenatal examination, essential medicines, and prominent display boards in local language were present in almost all SCs. Record-keeping practices were not uniform, and 14% of the SCs were not maintaining the record of early registration of antenatal care- and Integrated Management of Neonatal and Childhood Illness (IMNCI)-based assessment of children, whereas 26 SCs (90%) were not maintaining partograph charts. Health Management Information System (HMIS) data entry was uniformly complete, up-to-date, and accurate. In all the SCs, untied funds were used, while the Annual Maintenance Grant (AMG) utilization rate was less than 50%.

**Table 1:** Facility assessment of the subcenters (n = 29) IPHS: Indian Public health standards; IMNCI: Integrated Management of Neonatal and Childhood Illnesses; HMIS: Health Management Information System; ASHA: Accredited Social Health Activist; ANM: auxiliary nurse-midwife

Characteristics	Assessment		
Infrastructure	Good	Average	Poor
Condition of the subcenter building	12	15	2
Cleanliness and hygiene	18	10	1
Rooms used for providing services	17	12	0
Toilet availability and condition	21	5	3
24-hour water supply	20	1	8
Uninterrupted electricity supply	9	2	18
Logistics and supplies	Available and usable	Available but not usable	Not available
Equipment for antenatal examination	29	0	0
Essential medicines as per the IPHS	28	1	0
Tracking bags for routine immunization cards	15	14	0
Colored bags for biomedical waste disposal	29	0	0
Display of information, education, and communication material	24	5	
Record-keeping practices	Maintained	Not maintained	Not available
Early registration of antenatal care cases	25	4	
Partograph chart	0	3	26
Line listing of anemia cases	23	6	0
Eligible couple record	27	2	0
Routine immunization micro plan	27	2	0
IMNCI register	2	27	0
Mother and child tracking system work plan	0	0	29
HMIS completeness, timeliness, and accuracy	29	0	0
Training	21	8	0
Financial details	Available and utilized >80%	Available and utilized 80%50%	Available but utilized <50%
Untied funds	28	0	1
Annual maintenance grants	0	0	29
Monitoring and supervision activities done by	Good	Average	Poor
Medical officer	6	0	23
Lady health visitor	5	7	17
ASHA supervision by ANM	25	2	1

Service delivery

Services such as antenatal care, home-based postnatal care, childcare during immunization, family planning, and contraception were provided at all the SCs (Table 2). However, merely two-thirds of the SCs provided adequate services of essential examination in the first trimester (n = 21; 72%) and almost half in the second and third trimesters (n = 14; 48%). All births in the past year were through institutional deliveries, and only two (7%) SCs reported maternal death in the previous financial year. Five (17%) SCs provided average home-based postnatal care, and only 16 (55%) SCs provided adequate family planning services after detailed counseling. The vaccination coverage was >80% in most of the SCs (72%) and was between 50% and 80% of their target achievement in the other eight (27%) SCs. Only 30% (n = 10) confidently assessed and referred 0-2 month-old infants to a higher level healthcare facility.

**Table 2:** Service delivery characteristics related to maternal and child health observed during supportive supervision of the subcenters (n = 29)

Characteristics of the services provided to the	Good	Average	Poor
A. Antenatal women			
Registration of antenatal care during the first trimester	25	4	0
Essential examination in the first trimester	21	8	0
Essential examination in the second and third trimesters	14	15	0
Institutional delivery	29	0	0
Maternal death in the area	27	NA	2
Referral of high-risk pregnancies	24	2	3
Post-natal visits	24	5	0
Family planning services	16	13	0
B. Child health			
Vaccination coverage	21	8	0
Sick child (0–2 months) assessed and referred	10	-	19
Sick child (2 months–5 years) assessed and referred	27	-	2

Assessment of essential skills of the ANMs

OSCE test was used to assess the knowledge and practices of the ANMs (Table 3). The majority of the health workers (n = 27; 93%) demonstrated proper handwashing techniques, whereas only two-thirds (65%) of the women correctly measured BP as per the OSCE steps, and little less than half (48%) could correctly demonstrate Hb estimation with a hemoglobinometer. Most (89%) of them had sound knowledge regarding maternal and child healthcare practices and guidelines.

**Table 3:** Assessment of essential skills of health workers posted at the subcenters during baseline assessment visits (n = 29)

Scores obtained on objective structured clinical examination by subcenters	Good	Average	Poor
Handwashing technique	27	2	0
Blood pressure measurement with a sphygmomanometer	20	9	0
Hemoglobin estimation with hemoglobinometer	14	15	0
Detection of albumin/glucose in urine with Uristix	26	3	0
High-risk pregnancy identification	28	1	0
Cold chain maintenance	28	1	0
Diphtheria-pertussis-tetanus vaccine administration	27	2	0
Assessment of danger signs in the child	24	5	0

Overall assessment of the SCs

Table 4 summarizes the overall status of SCs based on the scores given. The SC score was maximum for HMIS, and availability of essential drugs and equipment, whereas the score was low in monitoring and supervision activities and maintenance of financial records.

**Table 4:** Categorization of subcenters (n = 29) based on supportive supervision parameters/standards SC: subcenter; N: number of questions in each domain, also equivalent to maximum obtainable score for that domain; SD: standard deviation; IEC: information, education, and communication; HMIS: Health Management Information System; ANM: auxiliary nurse-midwife

Parameter/standard (maximum obtainable score)	Number of SCs under different categories			Mean (SD, range)	Group score	
	Good	Average	Poor		Total possible score (N×29)	Total score obtained (%)
Infrastructure and general overview (n = 12)	13	14	2	8.24 (2.48, 3–12)	348	239 (68.6)
Essential functioning equipment (n = 6)	29	0	0	5.51 (0.50, 5–6)	174	160 (91.9)
Essential drugs availability (n = 2)	28	1	0	1.96 (0.18, 1–2)	58	57 (98.2)
Record-keeping practices (n = 28)	26	1	0	22.03 (2.24, 17–25)	812	639 (78.69)
Upkeep of financial details (n = 4)	0	29	0	2 (0, 0)	116	58 (50)
IEC activity (boards/posters) at SCs (n = 2)	24	5	0	1.82 (0.38, 1–2)	58	53 (91.3)
Provision of reproductive and child health services (n = 6)	16	13	0	4.55 (0.82, 3–6)	174	132 (75.8)
HMIS (n = 6)	29	0	0	6 (0, 0)	174	174 (100)
Training of ANM (n = 2)	21	8	0	1.72 (0.45, 1–2)	58	50 (86.2)
Knowledge and skills (n = 16)	28	1	0	14.68 (1.36, 11-16)	464	426 (91.8)
Monitoring and supervision (n = 4)	3	12	14	1 (1.16, 0–4)	116	29 (25)

## Discussion

This is among the few studies from India that aimed to assess the adequacy of SCs through SS activities. It mainly focused on assessing the necessary infrastructure and skills that help in effective service delivery by using a comprehensive tool based on IPHS [16]. Moreover, this study documented the knowledge and skill levels of the ANMs recruited under the National Rural Health Mission in 2005 (now renamed as National Health Mission) that prioritized human resources training but produced mixed long-term results [17]. Our study findings reflect the unmet need for supervision and the limited time that supervisors provide in delivering routine immunization services. This initial assessment depicts that most of the SCs still have average infrastructure, and there is room for improvement in record-keeping practices. The quality of service delivery depends mainly on the skills of health workers, which were average in most of the SCs. We stress on different aspects of SS like the regularity of visits by the local officials, mentoring and collaboration, and dialectic communication between supervisors and health workers.

In our study, all the SCs were providing services to more than 5,000 people, which is more than the IPHS norms for SCs, indicating a high burden on the existing SCs for service delivery. The results are similar to the study from Belagavi, Karnataka [18]. All the SCs had their own buildings; but in 2012-2013, only 50% SCs in Chandigarh and Panchkula were found to be working from their own premises [19]. Although ANMs are supposed to spend most of their time in the community, approximately two-thirds of their total time is spent in center-based activities (conducting an antenatal clinic, well-baby clinic, adolescent clinic, family welfare clinic, and Janani Suraksha Yojana registration). This necessitates the demands of a well-organized SC [20].

Well-organized SCs improve productivity and, hence, job satisfaction [21]. All the SCs had ANMs (either on a regular basis or contractual basis) and ASHAs, which are better than the rural health statistics report 2015, which depicted 85% occupancy of SCs by ANMs. By contrast, only 13.6% of the SCs had a male health worker (MHW). Moreover, significant gaps were observed in two other districts of Haryana, India, with the MHW post vacant in most of the places [19,22]. This perceived low priority given to MHW by the state governments might be due to their ill-defined job responsibilities. MHWs usually spend the majority of their time on the national malaria eradication program and health education activities [23]. In light of the current absence of these workers, further research may evaluate the functioning and effectiveness of the National Vector Borne Disease Control Programme (NVBDCP) activities.

Few SCs were providing adequate services on family planning and maternal and child health. The facilities were similar to those offered in other parts of India [20]. Moreover, the clinical skills exhibited by ANMs were less than satisfactory in most SCs, which is a cause of concern as they are usually the first point of contact at the periphery. Poor clinical skills lead to inappropriate service delivery and, hence, poor program outcomes. Moreover, Singh et al. observed inadequate use of services in the SCs of Madhya Pradesh [24]. Inadequate clinical assessment may lead to a false sense of well-being for most of the people who may need urgent medical care. In this situation, regular supervision using clearly defined and quantifiable indicators can improve service delivery considerably at a modest cost [25]. Another report documented that intense supervision led to a high provider performance, despite initial resistance and extreme variation in individual performance [26]. Financial grants were present only in the form of untied funds in most of the centers that were used entirely. This money is used for repair, payment for tenanted buildings, etc. Nandan et al. (2007-2008) observed that at most SCs, more than 90% of the untied fund is spent [27]. Round-the-clock facilities were not available in any of the SCs as none of the health workers were maintaining headquarters, a typical situation, as observed in another study as well [24]. However, recently, the Government of India has initiated the transformation of SCs into health and wellness centers that can provide essential medical services round the clock. These health facilities will act as a provider of holistic healthcare services package with zero cost to the patient [28].

The results of the skill assessment of ANMs were motivating as they knew the need and purpose of services delivered at the primary healthcare level. For example, most of the health workers knew about hypertension and diabetes mellitus, their implications, and how to measure them. The mean score was comparable with another study, although the skills were average when they demonstrated the process of BP measurement with a sphygmomanometer, Hb estimation with a hemoglobinometer, and detection of albumin/glucose in urine with Uristix [29]. The health workers were simultaneously taught regarding the processes that were observed to be wrong or missing during the OSCE examination, and they wholeheartedly welcomed it. We need to address the gaps in skills by providing on-job training and demonstrations to health workers at regular intervals.

The study shows that SS emergence paved the way not only for assessing performance but also for monitoring services, evaluating management, and ensuring proper working of health facility supply chains [30]. Moreover, the study has highlighted that budget constraint should not be blamed for the low performance of the SCs. Most of the parameters that can be improved (skill-building, training, record-keeping practices, and monitoring) are within the scope of the existing means and resources. Moreover, the success of the study can be assessed through the determination of its adherence to the SS principles described in the background section of this paper, such as handholding for quality improvements, strict use of validated tools, and skill assessment against set standards. However, this study had certain limitations as well. In addition to the small sample size, a mixed-method approach could have been used in the study to reveal different aspects regarding the deficiencies from various stakeholders and probable scope for the improvement of service delivery. We could not assess the long-term effect of SS, as it was a cross-sectional study. Future studies should aim to lay down the dashboard indicators that can objectively evaluate the healthcare delivery through SCs and revisit them after a while to look for sustainable changes.

## Conclusions

The present study has highlighted the role of SS in improving the delivery of services in healthcare. Implementation of SS is recommended within the framework of national health systems with similar institutional arrangements for health services organizations. We recommend putting in place supervision services that can provide complementary support mechanisms to health workers and supervisors alike for the overall achievement of universal health coverage. Supervision activities can be strengthened through streamlining supervision protocols to focus less on report checking and more on problem-solving and skill development.
